# Optimizing Boron Neutron Capture Therapy (BNCT) to Treat Cancer: An Updated Review on the Latest Developments on Boron Compounds and Strategies

**DOI:** 10.3390/cancers15164091

**Published:** 2023-08-14

**Authors:** Andrea Monti Hughes, Naonori Hu

**Affiliations:** 1 Radiation Pathology Division, Department Radiobiology, National Atomic Energy Commission, San Martín, Buenos Aires B1650KNA, Argentina; 2 National Scientific and Technical Research Council, Ciudad Autónoma de Buenos Aires C1425FQB, Argentina; 3 Kansai BNCT Medical Center, Osaka Medical and Pharmaceutical University, Osaka 569-8686, Japan; naonori.ko@ompu.ac.jp; 4 Institute for Integrated Radiation and Nuclear Science, Kyoto University, Osaka 590-0494, Japan

**Keywords:** boron neutron capture therapy, BNCT, targeted therapy, new boron compounds, strategies, radiobiological studies, theranostic compounds

## Abstract

**Simple Summary:**

Boron neutron capture therapy (BNCT) is a tumor-selective particle radiotherapy. It combines preferential boron accumulation in tumors and neutron irradiation. Throughout the history of BNCT, huge efforts have been made on the development of more efficient boron-carrying agents. We analyzed, in this review, those articles published between 2020 and 2023 reporting new boron compounds and strategies that were proved therapeutically useful in *in vitro* and/or *in vivo* radiobiological studies, a critical step for translation to a clinical setting. We also explored new pathologies that could potentially be treated with BNCT. We analyzed examples of newly studied theranostic boron agents that could improve significantly BNCT therapeutic effect. These radiobiological advances intend to solve those limitations and questions that arise during patient treatment in the clinical field, with BNCT and other therapies. An active communication between clinicians, radiobiologists, and all disciplines will improve BNCT for cancer patients, in a cost- and time-effective way.

**Abstract:**

Boron neutron capture therapy (BNCT) is a tumor-selective particle radiotherapy. It combines preferential boron accumulation in tumors and neutron irradiation. The recent initiation of BNCT clinical trials employing hospital-based accelerators rather than nuclear reactors as the neutron source will conceivably pave the way for new and more numerous clinical trials, leading up to much-needed randomized trials. In this context, it would be interesting to consider the implementation of new boron compounds and strategies that will significantly optimize BNCT. With this aim in mind, we analyzed, in this review, those articles published between 2020 and 2023 reporting new boron compounds and strategies that were proved therapeutically useful in *in vitro* and/or *in vivo* radiobiological studies, a critical step for translation to a clinical setting. We also explored new pathologies that could potentially be treated with BNCT and newly developed theranostic boron agents. All these radiobiological advances intend to solve those limitations and questions that arise during patient treatment in the clinical field, with BNCT and other therapies. In this sense, active communication between clinicians, radiobiologists, and all disciplines will improve BNCT for cancer patients, in a cost- and time-effective way.

## 1. Introduction

Cancer is one of the leading causes of death in the world. It is a large group of diseases, originated by abnormal cells of the body that start growing without control, going beyond their usual boundaries, invading neighboring tissues, and/or spreading to other organs. Cancer generates high economic costs and is a major burden on humanity. An increase in cancer cases of 47% (compared to 2020) across the world is forecast for 2025. These numbers reflect the imperative need for improving the strategies for cancer prevention, screening, diagnosis, and management [1,2].

Chemotherapy and/or radiotherapy have been widely used as the classical oncological treatments. However, important limitations have been reported related to their therapeutic effectiveness and the induced adverse effects in normal tissue. In this sense, new strategies are needed to overcome these issues [2,3]. For example, biological therapies like immunotherapy (immune checkpoint inhibitors, engineered cellular immunotherapies like chimeric antigen receptor (CAR)-modified T cells, among others) have gained great interest due to their relatively high therapeutic effectiveness and lower toxicity [2]. In the field of radiation therapies, charged-particle therapy like boron neutron capture therapy (BNCT), proton beam therapy, and carbon-ion radiotherapy have improved biological and physical dose distributions with higher relative biological effectiveness over conventional photon therapy [4,5]. 

Particularly, BNCT is a tumor-selective particle radiotherapy that combines the advantages of biological targeting and heavy ion radiotherapy [6]. It is a non-mutilating and organ-preserving therapy. It consists of one or at least two applications and has been useful to treat diffuse or irregularly shaped solid tumors [5]. BNCT is considered a binary treatment, as it combines the administration of a drug containing ^10^B, a non-radioactive isotope of boron which accumulates preferentially in tumor cells, with thermal or epithermal neutron beam irradiation [7,8]. 

**Figure 1:**
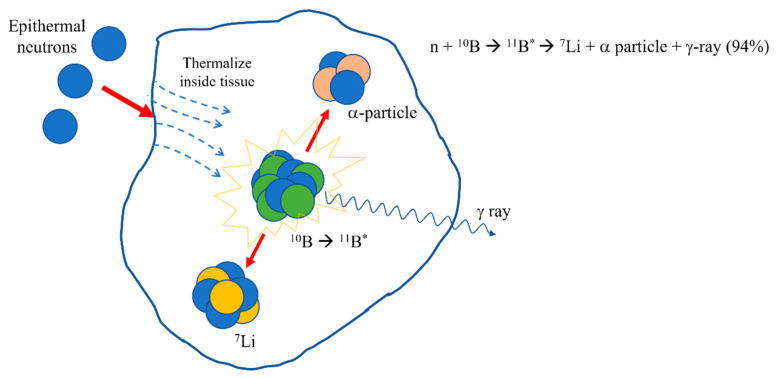
Schematic of the nuclear reaction between ^10^B atom and a thermal neutron. Epithermal neutrons are thermalized when they travel through the tissue. The high-LET particles deposit their energy within the cell where the reaction took place.

BNCT is a mixed-field radiation composed of ionizing radiation with different LET characteristics. The capture reaction of a thermal neutron by ^10^B results in a high linear energy transfer (LET) alpha particle (^4^He) and a recoiling lithium nucleus (^7^Li), which deposit their energy along a path of one cell diameter (approximately <10 μm), as shown in Figure 1. In this sense, high-LET particles will preferentially damage tumor cells, while preserving normal surrounding cells [9,10]. 

Apart from the tumor-specific boron dose component (high-LET alpha and lithium particles), there is a non-specific background dose that similarly affects both the tumor and the surrounding normal tissues [9,10]. Those BNCT protocols that could maximize the boron radiation component would increase tumor control while reducing possible radiotoxic effects in normal surrounding tissues [11]. As BNCT is characterized by its biochemical rather than geometrical targeting, it would be capable of treating not only the tumor but also undetectable micrometastases and foci of malignant transformation in field-cancerized tissue [4,12,13].

BNCT has improved patients’ quality of life, induced partial and complete responses, and prolonged survival. BNCT’s therapeutic efficacy with no significant toxicity has been proved for glioblastoma multiforme, melanoma, recurrent head and neck tumors, lung and liver metastases, mesothelioma, and extramammary Paget’s disease, e.g., [14,15,16,17,18,19,20,21]. The boron compounds approved for use in humans are mercaptoundecahydrododecaborate-^10^B, sodium borocaptate (BSH) and boronphenylalanine (BPA), principally employing nuclear reactors as neutron sources. The majority of BNCT-treated patients had no other treatment options at the time of irradiation. These clinical studies were/are currently being performed in the United States, Japan, European Consortium, Sweden, Italy, Finland, Argentina, China, and Taiwan (among others). Nowadays, huge efforts are devoted to the introduction of hospital-based accelerators rather than nuclear reactors. This innovation will increase significantly the number of patients that could be treated with BNCT [22]. Moreover, since 2020, Japan has been providing insurance-covered BNCT treatment for recurrent head and neck cancer [23]. 

Throughout the history of BNCT, huge efforts have been made on the development of more efficient boron-carrying agents. Although BPA and BSH have been therapeutically useful, there is room for improvement. Lists of important requirements for boron compounds in BNCT have been cited in many papers: (1) a low intrinsic toxicity, (2) a high tumor uptake (>20 µg ^10^B) and a low normal tissue uptake (tumor:normal tissue and tumor:blood boron concentration ratios of >3:1), and (3) rapid clearance from the blood and normal tissues with the maintenance of the boron concentration in the tumor during neutron irradiation [4,24,25,26]. A wide range of compounds have been developed, ranging from small molecules to boron-containing compounds modified with glucose, folic acid, amino acids, cell membrane-penetrating peptides, nucleoside and carbohydrate analogues, nucleotide borate esters, DNA intercalators, metallocarboranes, porphyrins, boron-charged antibodies, liposomes, nanoparticles, dual targets that reach tumor populations in normoxia and hypoxia, etc., e.g., [4,25,26,27,28,29,30,31,32,33,34,35]. Although they have not been used in clinical trials, they have paved the way to the design of more complex compounds that involve different functions/targeting capacities all in the same boron carrier. 

In this review, we made a summary of the most recent advances in BNCT radiobiological studies on new boron compounds, strategies, and pathologies. We analyze those articles published between 2020 and 2023 that included *in vitro* and/or *in vivo* radiobiological studies, a critical step to decide if these findings could be translated to a clinical setting. These studies have been based on limitations and questions that arise in the clinical field, not only in BNCT but also in other cancer therapies. 

## 2. BSH, BPA, GB-10, and Boric Acid: Brief History and Optimized Delivery Strategies

In 1968, a team led by Dr. Hatanaka began administering BNCT to patients with malignant brain tumors using BSH [36]. The compound BSH has an excellent ability in transporting ^10^B (it carries 12 boron atoms) and has a high water solubility. The blood–brain barrier (BBB) prevents BSH from entering the brain tissue, but in malignant brain tumors, this barrier is dysfunctional, allowing BSH to enter and accumulate, resulting in a significant concentration difference. The results obtained by Dr. Hatanaka were favorable and suggested the efficacy of BNCT [37]. 

In the early stages of BNCT research, it was predicted that BPA would replicate endogenous phenylalanine’s properties and preferentially boost its uptake in malignant cells with an excessively high melanin concentration. In 1987, a group led by Dr. Mishima were the first to clinically use BPA to treat a patient with malignant melanoma and later used it for patients with recurrent malignant glioma. BPA/BNCT is a possible treatment for many pathologies, as BPA uptake is through L-type amino acid transporter 1 (LAT1) which is overexpressed in many tumor cells [36,38,39,40]. Moreover, BPA could be able to reach infiltrative glioma cells in the normal brain parenchyma where the BBB is still intact, as BPA crosses the blood–brain/–tumor barrier via LAT1 [33,41]. With the introduction of BPA, BNCT may be referred to as a technique for selective cancer cell treatment.

However, BPA has limitations related to solubility and capacity to carry boron atoms (it carries only one boron atom). The phenylalanine hydrophobic structure and neutral charge at physiological pH results in intermolecular salts that are difficult to dissolve. At the beginning of BPA clinical application, BPA–hydrochloride solution at low pH was used to overcome this aspect. Afterwards, Dr. Yoshino and coworkers found that sugars including fructose can form complexes with BPA and increase its water solubility. Nowadays, fructose is principally used as a BPA solubilizer [38]. 

Only the isotope of ^10^B of the naturally occurring boron can capture thermal neutrons. To produce the boron compounds utilized in BNCT, high-purity ^10^B is required. Only a few countries, such as Japan and the US, have factories that can produce enriched ^10^B in large quantities. In March 2020, the Japanese Ministry of Health, Labour and Welfare approved STEBORONINE 9000 mg/300 ml (borofalan (^10^B): boronated phenylalanine, Stella Pharma Corporation, Osaka, Japan) for use in BNCT treatment [42]. 

**Figure 2:**
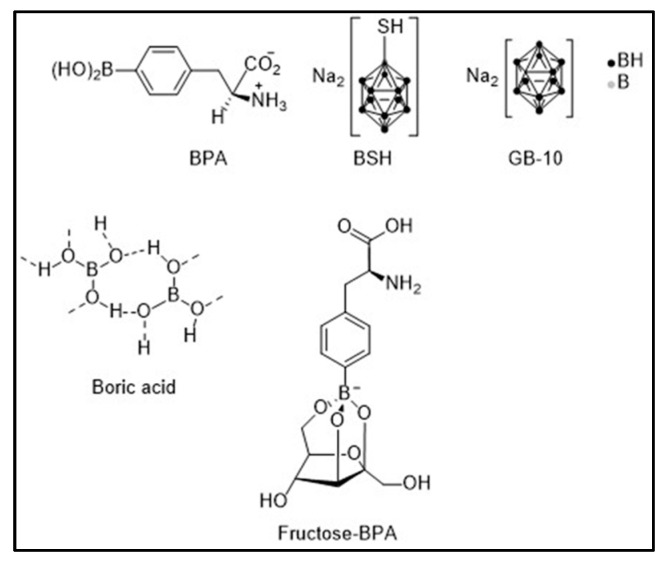
Chemical structures of BPA, BSH, GB-10, boric acid, and fructose–BPA.

BPA and BSH have been studied alone, in combination, or administered jointly with boric acid and decahydrodecaborate (GB-10). These are low molecular weight boron compounds like BPA and BSH. Boric acid and GB-10 have diffusive characteristics. Boric acid was one of the first compounds used in BNCT clinical trials in the 1950s–1960s. GB-10 was once approved for use in patients by the US Food and Drug Administration [25,43]. Figure 2 shows the chemical structure of each of the abovementioned boron compounds.

Radiobiological studies have shown that strategies that could increase boron uptake in the tumor, enhance tumor/blood and tumor/normal tissue boron concentration relations, and increase retention during irradiation and microdistribution in the tumor (targeting all tumor cell populations, including stem cells) using these well-known boron compounds increased BNCT’s therapeutic effect and reduced its toxicity. Different tumors have been explored, like glioblastoma, squamous cell carcinoma from head and neck cancer, prostate, pancreas, thyroid, breast, gastric, oral cancer, liver metastases, hepatoma, osteosarcoma, and lung metastases, e.g., [44,45,46,47]. 

In the next sections, we will describe the most recent BNCT *in vitro* and *in vivo* radiobiological studies on new boron compounds, strategies, and pathologies between 2020 and 2023. 

### 2.1. Newly Studied Pathologies for BNCT 

Cervical cancer is the fourth most frequent female cancer worldwide. Surgery or irradiation is the primary treatment, although radiotoxicity is dose-limiting. If tumors recur, re-irradiation is not possible due to significant toxicities. Additionally, tumor response to radiotherapy depends on the tumor histological type, as cervical adenocarcinoma is more radioresistant than squamous cell carcinoma. Terada et al. [48] studied the possibility of treating these two types of cervical cancers with BNCT. They demonstrated that LAT1 was expressed in human cervical squamous cancer and cervical adenocarcinoma tumor cells. Boron uptake was therapeutically useful in both cell lines. Although boron concentrations in cervical cancer cells decreased rapidly, BNCT mediated by BPA decreased the survival fraction of both cell lines. Finally, in an *in vivo* ectopic cervical cancer model (induced with both cell lines) tumor boron uptake was therapeutically useful, independently of the histological type, and was greater than boron concentration in other tissues. The authors mentioned, based on these results, the importance to study BNCT in an *in vivo* orthotopic model, to evaluate the safety of dose-limiting tissue such as normal uterine and intestines. They also mentioned another limitation: thermal neutron beams would not reach the cervix. However, BNCT may be able to treat metastatic lesions in more superficial organs and vaginal and vulvar cancers. 

Colorectal cancer is the fourth most common cancer worldwide. Multimodality treatment of rectal cancer consists of surgery and chemoradiotherapy. Although these therapies improve patient outcomes, some patients exhibit local recurrences that affect prognosis and decrease quality of life. Treatment for recurrences is limited, for example, most patients with local recurrence of rectal cancer have previously undergone irradiation to the pelvis, and re-irradiation in these cases has been discouraged because of toxicity to normal tissues [49]. In this sense, new therapies are necessary, and BNCT has been proposed as a possible strategy for this malignancy. Arima et al. [50] studied BNCT mediated by BPA in mice injected with colorectal cancer cells in the pelvic retroperitoneum. They reported a significant increase in animals’ survival without signs of diarrhea, intestinal hemorrhage, or intestinal perforation after irradiation. Although the bladder exhibited the highest BPA concentration (BPA is removed by the urinary system), pathological examination showed no significant differences between irradiation only and BNCT groups. Despite this positive result, they did suggest that bladder and uterus have to be excluded from the irradiation field. This study has the limitation that the authors did not evaluate intestine after BNCT and that pelvic CRC in this mouse model is nearer to the body surface compared with that in humans. In this sense, neutron beam energy and penetration should be carefully studied when treating pelvic CRC like in cervical cancer, as we mentioned before. 

Yoshimura et al. [51] suggested the use of BPA/BNCT for primary central nervous system lymphoma (PCNSL). PCNSL is a new lymphoma entity. PCNSL has a very high response rate to initial therapy, like chemotherapy and autologous stem cell transplantation [52,53]. However, almost all patients relapse and recur, and there are no effective and safe treatments in these cases. In this sense, new therapies are needed, and BNCT could contribute to curing this illness. Previous studies showed that PCNSL cells express high levels of LAT1 [54], making them feasible for being treated with BPA/BNCT. Yoshimura et al. [51] studied BNCT mediated by BPA in a mouse central nervous system (CNS) lymphoma model, in which tumors developed after implanting lymphoma cells in the brain. They demonstrated boron uptake *in vitro* and *in vivo*. BPA/BNCT induced high cytotoxicity and prolonged survival.

In current BNCT clinical trials, BPA has to be administered at an extremely high dose (500 mg/kg) and requires a few hours to accumulate at tumor sites. To address this limitation, Yoneyama et al. [55] designed the carbohydrate mimetic peptide IFLLWQR (IF7), conjugated with BPA and BSH, that targets annexin A1 (Anxa1) within minutes of injection. Anxa1 is essential for tumor vascularization and has functions in immunity. Annexin family proteins localize on the surface of tumor endothelial cells in several tumor types and enter the cells by endocytosis. The authors studied an ultralow dose administration protocol (10–20 mg/kg) of IF7C(^10^BPA)RR or IF7K(^10^BSH)RR to treat bladder tumor-bearing mice. They observed rapid ^10^B accumulation in tumor tissues and significantly suppressed tumor growth with no apparent side effects. BNCT mediated by IF7-^10^B drug induced tissue necrosis and infiltration of CD8α-positive lymphocytes, demonstrating that BNCT induced an immune response by the host against tumor cells, triggering a strong cytotoxic reaction. 

In the field of breast cancer, it is known that 70% of these tumors metastasize to bones, inducing bone destruction and thus affecting patient quality of life. Although there are alternative therapies to treat these metastases, they exhibit limitations. For example, curative doses of X-rays cause fatal radiation damage to normal bones. In this sense, Andoh et al. [56] demonstrated that BNCT is capable of inducing tumor growth inhibition, while preventing bone destruction and preserving bone health. They established a bone metastasis model for breast cancer by implanting a human breast cancer cell line into the tibia of the left or right hind leg of nude mice. They observed an extremely high level of BPA uptake. After BNCT, the tumor decreased in size without damage to the normal surrounding bone tissue. Moreover, BNCT induced a remodeling of bone at the bone metastasis site for those cases without pathological fracture. 

### 2.2. BPA-, BSH-, Boric Acid-, and GB-10-Mediated BNCT, Derivatives, and Strategies to Enhance BNCT Therapeutic Effect 

#### 2.2.1. BPA and BSH 

Radiation produces cell membrane effects that could increase the entrance of different molecules to the cell. Paracrine ligand expression, protease activity, and receptor expression were associated with an increase in the entrance of adenovirus and antisense oligonucleotides [57,58,59]. BSH was previously studied combined with a 5 Gy dose of γ-rays. This strategy increased animal survival and induced tumor complete responses, with no toxicity [60]. In 2021, Lin et al. [61] combined a low dose of γ-radiation (0.1 Gy, LDR) with BPA in an orthotopic human oral squamous cell carcinoma-bearing animal model. They observed that LDR increased BPA accumulation in tumors by 52.2%. Tumor/normal tissue and tumor/blood ratios were enhanced from 3.77 to 5.31 and from 3.47 to 4.46, respectively. They improved BNCT’s therapeutic effect with no associated toxicity. They demonstrated a 100% overall survival rate and complete responses increased to 83% vs. 50% in the BNCT only group.

Another strategy that increased boron accumulation in the tumor and BNCT’s therapeutic effect is the combination of BPA with the radiosensitizer sodium butyrate (NaB) [62]. It is the sodium salt of butyric acid (a fatty acid). Previously, its antitumor and radiosensitizing activity was shown to be associated with gamma radiation and BNCT *in vitro*. Perona et al. [62] studied the effect of combining BPA/BNCT with NaB in an *in vivo* ectopic model of human thyroid cancer. Biodistribution studies showed an increase in boron uptake. When they evaluated the expression of LAT transporters, the administration of NaB activated LAT2, 3, 4 genes, apart from LAT1. BPA/BNCT + NaB enhanced tumor complete remissions versus the BNCT only group.

Abnormal angiogenesis and rapid growth lead to insufficient blood flow and hypoxic regions with poor oxygen supply. Hypoxia-inducible factor 1α (HIF-1α) accumulates as an adaptive response to hypoxia in hypoxic cells to survive. Harada et al. [63] reported that BNCT’s therapeutic effect was attenuated in hypoxic glioblastoma and head and neck cancer cells. To understand this effect, they performed knockdown studies aimed at HIF-1α and showed that HIF-1α suppresses LAT1 expression in hypoxic tumor cells. With this in mind, the authors combined BNCT with an HIF-1α-targeting inhibitor called 3-(5′-hydroxymethyl-2′-furyl)-1-benzylindazole (YC-1) and proved that YC-1 sensitized the antitumor effects of BNCT in both glioblastoma and head and neck cancer cells cultured in hypoxic conditions. Moreover, Sanada et al. [64] contributed to these studies as they observed that hypoxia improved cell survival after neutron irradiation with BPA, but not with BSH.

It is known that boron targeting, uptake, and retention are very important factors that condition BNCT’s therapeutic effect. However, tumor local treatment sometimes is not enough to cure a cancer patient, as metastases are the main cause of death. In this sense, strategies that could increase possible effects of BNCT at distant sites of the primary tumor are necessary. It was demonstrated that ionizing radiation can induce immunogenic cell death that may trigger a cytotoxic immune response against not only the primary tumor but also its metastasis. This phenomenon is called the abscopal effect and has been reported after standard radiotherapy and after BNCT [65,66]. Recent publications mentioned that BNCT induces immunomodulatory effects that may contribute to tumor growth inhibition [55,67]. In this sense, strategies that could enhance BNCT’s immunomodulatory effect would contribute to inhibiting the growth of the primary tumor and its metastasis. Previous studies showed that genetically modified microorganisms can stimulate the immune system and cause tumor regression. One example is bacillus Calmette–Guérin (BCG), approved as the gold-standard treatment for non-muscle invasive bladder cancer [68]. Trivillin et al. [69] proposed, for the first time, the study of the local, regional, and abscopal effects of BNCT mediated by BPA combined with bacillus Calmette–Guérin (BCG) as an immunotherapy agent. For that aim, BDIX rats were first injected subcutaneously with syngeneic colon cancer cells in the right hind flank (irradiated leg, local BNCT effect). Two weeks post-irradiation, colon cancer cells were injected subcutaneously in the contralateral left hind flank (non-irradiated leg, abscopal BNCT effect). Metastatic spread to tumor-draining lymph nodes was analyzed as an indicator of regional effect. BNCT and BNCT + BCG induced a highly significant local antitumor response and a significant abscopal effect in the contralateral non-irradiated leg. Particularly, the BNCT + BCG group showed significantly less metastatic spread to tumor-draining lymph nodes. 

Fukuo et al. [33] developed BPA–amide alkyl dodecaborate (BADB), a boron carrier with 13 boron atoms per molecule. This compound showed high accumulation in three different glioma cell lines, melanoma, and squamous carcinoma (human and mouse lines). They then evaluated boron concentration in an *in vivo* glioblastoma model, comparing BPA (administered intravenously) and BADB administered by convention enhanced delivery (CED). CED allows a direct infusion of BADB into a tumor using pressure-driven bulk flow without having to pass the drug through the blood–brain barrier. The BADB group exhibited significantly higher boron concentration in tumors together with high tumor/normal brain and tumor/blood ratios versus BPA. Finally, BNCT *in vivo* showed no differences in the survival time of the animals treated with BADB/BNCT vs. BPA/BNCT. However, when they combined BADB (CED) and BPA (iv), they had longer survival times and the highest percentage of increased life span. 

As we mentioned before, one of the most important limitations of BPA it is poor retention in tumors. BPA quickly and efficiently accumulates in the tumor, but gradually decreases during irradiation of thermal neutrons. This constraint makes necessary a BPA infusion during irradiation, with the possibility of dislodgement of the injection needle in the middle of the irradiation and the need for complicated settings. This effect is due to an antiport mechanism: when LAT1 imports an extracellular BPA into the cytosol, it also exports an intracellular substrate. If the extracellular BPA concentration is decreased, intracellular BPA should be exchanged with an extracellular amino acid. Previous studies have shown the possibility of a pre-administration of substrates of LAT1, and this strategy enhanced tumor accumulation of BPA [70]. In Nomoto et al. [38], this limitation was overcome by complexing a poly(vinyl alcohol) with BPA (PVA-BPA). PVA-BPA is internalized into the cell through LAT1-mediated endocytosis, an active type of transport through which large extracellular material enters the cells that could not be taken up by diffusion or channels and transporters [71]. The advantage of this mechanism is that it slows the untoward efflux of PVA-BPA from the intracellular region, increasing boron compound retention inside the cell. Nomoto et al. [38] compared boron uptake and retention of PVA-BPA versus fructose-BPA in two *in vivo* ectopic models: human pancreatic adenocarcinoma and colon cancer. They showed in both animal models a significantly higher accumulation and longer retention in the tumor with quick clearance from bloodstream and normal organs. As to the therapeutic potential of BNCT evaluated in these *in vivo* models, fructose-BPA and PVA-BPA suppressed tumor growth, however, PVA-BPA showed a significantly higher antitumor activity for a longer period of time.

Another strategy that could take advantage of endocytosis is mesoporous silica-based nanoparticles. They exhibit low toxicity, high stability in the bloodstream, and, from a chemical point of view, their highly specific and large surface area makes them ideal substrates for attaching drugs. Tamanoi et al. [39] studied biodegradable periodic mesoporous organosilica (BPMO) nanoparticles loaded with BPA (BPA-loaded BPMO). These nanoparticles have enhanced degradation under reducing conditions such as that encountered inside cells. The authors demonstrated an efficient uptake into human ovarian cancer cells, with perinuclear localization. *In vivo* biodistribution and BNCT studies were performed in a chicken egg human ovarian tumor model, a versatile animal model that has been used for the characterization of nanoparticles. BPA-loaded BPMO nanoparticles significantly accumulated in tumors and BNCT mediated by BPA-loaded BPMO demonstrated a significant tumor growth inhibition. Laird et al. [72] used the same strategy, but loading BSH. This study was performed in human ovarian cancer spheroids. They demonstrated an efficient uptake by cancer cells with perinuclear accumulation. BSH-BPMO loaded spheroids were completely destroyed upon neutron irradiation versus tumor spheroids loaded with BSH or BPA, which exhibited significantly less spheroid shrinkage.

Macropinocytosis is an endocytic pathway that mediates the incorporation of non-selective extracellular material via large endocytic vesicles. This mechanism supports cancer cell metabolism through the uptake of nutrients. Cancer cells are known to display heightened macropinocytosis, allowing them to survive and proliferate despite the nutrient-scarce conditions of the tumor microenvironment [73]. The epidermal growth factor receptor (EGFR) is a key regulator in cell proliferation, differentiation, division, survival, and cancer development [74]. Between other molecules, EGFR has been implicated in macropinocytosis in tumor cells [75]. Nakase et al. [76] proposed an experimental “cassette” binding BSH to a Z33 peptide (Z33-DB), capable of binding to an antibody for receptor targeting on cancer cells. In this study, Z33-DB was complexed with cetuximab. Cetuximab is a monoclonal antibody that binds to EGFR, approved as a first-line treatment for many cancers. EGFR activation induces macropinocytosis that internalizes the Z33-DB compound. *In vitro* BNCT studies mediated by Z33-BD plus cetuximab induced an effective cell-killing effect in a human epidermoid carcinoma.

Liposomes and polymers were also proposed as efficient nanocarriers of BPA and BSH. One of the mechanisms by which liposomes accumulate in the tumor is by the enhanced permeability and retention effect (EPR). The EPR effect is a universal patho-physiological phenomenon and mechanism in which macromolecular compounds beyond a specific size accumulate in the tumor vascularized area. Tumor vessels are highly permeable to macromolecular compounds and, after entering tumor tissue, these compounds are entrapped inside the tumor for a prolonged period. The EPR effect acts in tumors with strong irregular neovascularization with abnormalities in tumor blood vessels, when there is an elevated expression of inflammatory factors and the lack of an efficient drainage of lymphatic systems in solid tumor tissue. However, this effect exhibits some limitations that could be overcome with specific molecular affinities [77]. Yanagie et al. [78] studied a ^10^BSH-entrapped transferrin-conjugated polyethylene glycol liposome constructed with distearoyl-boron lipids (TF-PEG-DSBL). This construction, apart from accumulating in the tumor by the EPR effect, binds to cancer cells and is internalized by receptor-mediated endocytosis. The authors, in an *in vivo* hepatic tumor model in rabbits, demonstrated that ^10^BSH-TF-PEG-DSBL liposomes increased boron retention in the tumor and suppressed tumor growth without pathological damage in normal hepatocytes after BNCT. 

As to polymers, for example, Fujimura et al. [79] studied a poly-arginine peptide (polyR) conjugated with BSH. BSH-polyR directly binds to the CD44 cell surface molecule for cellular uptake. CD44 is a stem cell-associated marker in various types of cancer. In this study, they demonstrated that BSH-polyR/BNCT successfully induced cell death specifically in high-CD44 glioma, breast, and pancreatic cancer cells. When BSH-polyR is inside the cell, it interacts with translation-related proteins via the BSH or polyR parts. BSH is capable of binding to poly(A)-binding protein 1 (PABP1), a multifunctional key protein with an important role in the translation process, while the other translational proteins were shown to interact with the polyR peptide. Thus, BNCT mediated by BSH or BSH-polyR might induce selective cell damage through damaging of the cell translational machinery. 

Fujimura et al. [79] is an example on how omics research, particularly in this case genomics analyses, paves the way to more personalized boron compounds, increasing the efficacy of BNCT. The authors analyzed the expression levels of CD44 in a clinical dataset and found that BSH-polyR might be suitable for certain types of malignant tumors. For example, BSH-polyR appeared tumor-selective for glioblastoma patients, as CD44 expression was not typically detected in normal brain. Moreover, BSH-polyR might be useful to treat patients with low expression levels of LAT1 transporters, thus not being candidates for BPA/BNCT. Transcriptomics and proteomics technologies would help to detect these types of patients. Finally, this strategy would increase patient survival after BNCT, as this compound targets CD44 stem cells that are responsible of recurrences after treatment. 

Michiue et al. [80] presented self-assembling A6K peptide nanotubes (peptide drug delivery system) as boron carriers. Their published preparation technique is simple: they mixed A6K (six alanine –A- residues and one lysine –K-) with BSH. A6K functions as a small interfering RNA (siRNA) delivery tool, targeting intracellular transduction. They showed that A6K/BSH was localized in the perinuclear region and in endosomes of human glioma cells. They reported that, with this derivative, boron uptake was almost 10 times higher than BSH alone and that BNCT mediated by A6K/BSH inhibited glioma cell proliferation. 

#### 2.2.2. GB-10 and Boric Acid

GB-10 has been well studied in the hamster cheek pouch oral cancer model. GB-10 is a diffusive agent, its uptake in tumor is low and its microdistribution in the tumor is preferentially in tumor stroma [44,81]. However, unexpectedly, BNCT mediated by GB-10 showed significant therapeutic effect on tumors in the hamster cheek pouch oral cancer model, by destroying selectively tumor blood vessels with no significant toxicity in dose-limiting tissues [44]. 

Strategies that could increase GB-10 uptake by the tumor and improve its microdistribution would enhance BNCT’s therapeutic effect. Electroporation (EP) permeabilizes the cell membrane by applying an electric field, allowing the passage of molecules into the cytosol. It is approved for use in humans with head and neck cancer and veterinary patients with oral and maxillofacial tumors. This technique increased boron uptake and boron homogeneity in oral tumor-bearing hamsters [82]. In 2023, Olaiz et al. [83] showed that EP increased significantly the therapeutic effect of BNCT mediated by GB-10 in the hamster cheek pouch oral cancer model, with no severe toxicity in the dose-limiting tissue. Tumor response reached 92% in the EP combined GB-10/BNCT group vs. 48% in the GB-10/BNCT alone group. Moreover, complete remissions increased up to 46% in the EP + BNCT group vs. 6% in the BNCT only group. EP and BNCT trigger immunogenic cell death. In this sense, the authors proposed that EP + BNCT + immunotherapies could be an excellent approach to offer a systemic therapy to patients suffering from diffuse tumors and/or metastases.

Hsu et al. [84] studied the therapeutic efficacy of BNCT mediated by boric acid (BA) to treat osteosarcoma (OS). Previous *in vivo* and *in vitro* studies showed that BA has a strong affinity for hydroxyapatite in the bone, because it binds to the cis-hydroxy groups. OS is the most common bone tumor and has been shown to be resistant to radiation therapy. In an orthotopic rat model of OS, BA-mediated BNCT effectively controlled tumor growth, reduced osteolysis, and induced bone healing. OS is an interesting target for BNCT, as it is mostly found in long bones, principally in the legs and sometimes in the arms. Since no critical organ is close to the long bone, the affected region can receive a high dose of irradiation. OS cells are characterized by the formation of calcified tissues from osteoids. BA accumulates more in the osteoids of tumor tissues than in the soft tissues. Additionally, the vasculature of a tumor is different from that of normal tissue, and it may take up more BA than normal vasculature. Therefore, when an OS undergoes neutron irradiation, the vasculature may suffer more damage than that of normal tissue, reducing BNCT’s induced toxicity. 

BA was also proposed for the treatment of hepatocellular carcinoma (HCC) [85]. BPA and BSH were not suitable boron compounds for the treatment of this disease, due to a low tumor/normal liver ratio and high boron accumulation in the nearby pancreas. In 2022, Huang et al. [86] explored if BNCT mediated by BA is also useful to eliminate radioresistant HCC cells *in vitro*. They demonstrated that, under the same dose of γ-ray, BNCT eliminated radioresistant HCC, increasing the number of DNA double strand breaks, impeding DNA reparation, and inducing cell arrest in G2/M phase and apoptosis. This is an important result, as radioresistance is one of the most important limitations of radiation therapy. 

Although boric acid has been proved useful for BNCT, previous studies showed that high boric acid concentrations induced toxicity in animal models. In this sense, Wu et al. [87] enhanced BA internalization by combining it with nanodrugs. They employed boric acid-containing chitosan/alginate/polyvinyl alcohol nanoparticles (BA-capNPs). These boric acid nanocarriers decreased toxicity by reducing exposure to boric acid and avoiding renal excretion due to an increase in the size of the molecule. In this study, they chose alginate as the drug carrier because of its characteristics related to stability, biocompatibility, and biodegradability. To avoid BA leakage from nanocarriers (due to its small size) polyvinyl alcohol (PVA) was used to crosslink with BA to form nanoaggregates. Chitosan was coated on the BA-capNPs to reveal positive charges that increase the affinity toward cancer cells compared with normal cells. BNCT mediated by BA-capNPs was tested in oral squamous cell carcinoma cells. BA-capNPs significantly reduce the cytotoxicity by 12-fold compared with BA and increase the killing efficacy of tumor cells by 2.8-fold compared with pure BA. 

Another strategy reported to enhance BA uptake is reported by Islam et al. [88]. They studied SGB-complex that consists of a water-soluble synthetic polymer, styrene–maleic acid copolymer (SMA) conjugated with glucosamine (SG), which formed a stable complex with BA. It is known that BA could form a complex with the cis-dihydoxyl groups of glucose, affecting the glycolytic pathway. This effect of BA would be more important in hypoxia, as energy production depends principally on glycolysis, the well-known Warburg effect. Hypoxia is a common state in large tumors [89]. Based on this knowledge, the authors demonstrated that SGB-complex by itself suppressed glucose uptake and cytotoxicity and it was taken up by tumor cells faster than free BA *in vitro*. *In vivo* SGB-complex binds with albumin and accumulated 10 times more in tumor than in normal organs. They reported that in tumor acidic conditions, BA was liberated from SGB-complex, inhibiting glycolysis and suppressing tumor growth *in vivo*. When they studied BNCT mediated by SGB-complex, they found a significantly enhanced therapeutic effect on human oral cancer cells and in squamous cell carcinoma-bearing mice. 

Hypoxia is associated with poor prognosis and is one of the key components that affect the cellular expression program and lead to conventional radiotherapy and chemotherapy resistance [90]. A recent study mentioned that hypoxia also could induce immunotherapeutic failure, as the prevalence of immunosuppressive populations within the hypoxic tumor microenvironment can confer tumor cell resistance to immune checkpoint inhibitors (ICPIs) and chimeric antigen receptor (CAR) T cells. In addition, hypoxia-triggered angiogenesis can also cause immunosuppression [91]. In this way, SGB-complex-mediated BNCT would be an interesting approach for those large tumors resistant to conventional therapies, taking advantage of their low pO2 characteristics to increase its therapeutic effect [88]. 

In Table 1, we summarize all the findings described in Section 2.1 and Section 2.2.

## 3. Development of New Boron Carriers

### 3.1. Targeting Cellular Structures and Relevant Molecules

Different cellular structures and cell molecules have been described as possible targets of boron-loaded compounds. DNA is the main target of radiation. It is known that the high-LET particles released after the boron neutron reaction induce direct damage in DNA, causing irreparable double strand breaks [92,93]. If DNA damage is not repaired, cell cycle arrest and different mechanisms of cell death could be activated. Apoptosis, necrosis, autophagy, or mitotic catastrophe has been described in different cell lines and *in vivo* models after BNCT [47,94,95,96]. In this sense, the development of boron compounds that could target DNA and/or the cell nucleus is in the spotlight. Novopashina et al. [97] published a mini review on the recent advances in the development of boron-loaded nucleic acids. For example, synthetic oligonucleotides (like antisense DNA, siRNA) have specific interaction with DNA and RNA in the cytoplasm or nucleus, and this characteristic makes them interesting as platforms to construct boron carriers. In this line, Kaniowski et al. [98] studied, in different cell lines, antisense oligonucleotides towards EGFR RNAm decorated with boron clusters. EGFR protein is involved in the development and spread of cancer and also contributes to chemotherapy and radiotherapy resistance. This construction has an antitumor effect *per se*, as it inhibits EGFR gene expression, while serving as a boron compound for BNCT. 

A strategy to target the nucleus was reported by Chen et al. [6]. They studied a multifunctional nanoliposome delivery system in a glioma mouse model that combines nuclear targeting with immunotherapy strategy, calling this a “boron neutron capture immuno-chemotherapy”. The authors designed DOXCB@lipo-pDNA-iRGD, a multifunctional nanoliposome that combines: (1) an internalizing RGD (iRGD), a peptide that targets a specific integrin expressed in different tumor cells and tumor neovascularization; (2) the nuclear tropism of doxorubicin (DOX). DOX is a widely used clinical first-line anticancer drug. It has antitumor activity, based on its ability to intercalate into the DNA helix and/or bind covalently to DNA replication and transcription proteins. It also has the capacity of inducing free radicals due to metabolic activation [99]; (3) CD47 blocking immunotherapy, i.e., a CD47 gene targeting a CRISPR–Cas9 gene knockout plasmid. CD47 is considered the “don’t eat me” signal, as it prevents phagocytosis of tumor cells. CD47 blocking by gene editing activates macrophage-mediated phagocytosis. Additionally, DOX is considered a “double-edge sword” due to its toxicity in non-targeted tissues [99]. With this multifunctional nanoliposome, DOX would be directed to tumor cells, sparing normal tissues. 

Apart from targeting DNA, evidence has shown that targeting mitochondria would increase the tumoricidal efficacy of radiation [100,101]. In this field, Kashiwagi et al. [102] focused on the 18 kDa translocator protein (TSPO) expression in glioblastoma. TSPO is a five-transmembrane domain protein located in the outer mitochondrial membrane. It was found that the higher the TSPO expression, the higher the severity of gliomas. Moreover, positron emission tomography (PET) studies with TSPO were able to predict the prognosis of patients with recurrent gliomas. In this sense, they studied DPA-BSTPG, a dodecaborated compound targeting TSPO alone and combined with BPA administration, in a glioblastoma *in vitro* and *in vivo* model. They demonstrated that the combination of these two boron compounds was capable of treating both the tumor and BPA-refractory glioma cells. 

Another reported target was the cytoskeleton in a glioblastoma cell line, studied by Belchior et al. [103]. Carboranylmethylbenzo[b]acridone (CMBA) is among the different proposed boron delivery agents that have shown promising properties due to its lower toxicity and important cellular uptake in human glioblastoma cells. CMBA can target cellular components mainly at the membrane and cytoskeleton but without a cytotoxic effect, a main requisite for the design of new BNCT agents. In particular, the results obtained for CBMA reinforce radiobiological effects demonstrating that damage is mostly induced by the incorporated boron, with negligible contribution from the culture medium and adjacent cells. 

Apart from these cellular organelles and structures, boron compounds aimed at targeting important proteins in tumor cells that play important roles in cancer cell proliferation and formation of metastases have been designed [104,105]. 

### 3.2. Nanobiocarriers

Changing to possible nanobiocarriers of boron agents, serum albumin has demonstrated significant results on tumor delivery and therapeutic effect. Serum albumin is of interest because of its capacity to bind to ligands to carry different molecules and it accumulates in tumors due to the combination of leaky and abnormal blood vessels, by the enhanced permeability and retention (EPR) effect. In addition, specifically the gp60 receptor located on the endothelial cell surface is responsible for transcytosis of albumin through the endothelium of blood vessels. Another reported mechanism is via the secreted protein acidic and rich in cysteine (SPARC) that promotes the accumulation of albumin in tumors because of its high binding affinity to albumin. Finally, the uptake of extracellular proteins is increased in the tumor, with albumin being the major nutritional source for the tumor [106,107]. 

Kikuchi et al. [106], Kashiwagi et al. [41], and Monti Hughes et al. [107] studied maleimide-functionalized closo-dodecaborate conjugated to bovine serum albumin (MID:BSA) or human albumin (MID:AC) in three different *in vivo* tumor models, a subcutaneous colon tumor model in mice, an orthotopic glioblastoma model in rats, and a carcinogen-induced oral cancer model in hamsters (respectively). They demonstrated a significant boron accumulation in the tumor and a significant therapeutic effect of BNCT, without associated radiotoxic effects. 

To improve MID-albumin targeting to tumor cells, Kawai et al. [108] studied the potentiality of combining MID:BSA with cyclic RGD. This peptide (mentioned previously in this review) has a great capacity to target tumor cells and tumor neovascularization and angiogenesis [109]. In this study, they improved tumor boron uptake compared to MID:BSA, in two different subcutaneous tumor models, to study glioblastoma and ectopic colon cancer. Particularly, in the glioblastoma xenograft tumor model, they demonstrated an enhancement of BNCT’s therapeutic effect when mediated by cRGD:MID:BSA compared to MID:BSA/BNCT. This year, Tsujino et al. [110] published their studies of cRGD:MID:AC in an orthotopic glioblastoma model. They demonstrated an enhancement in the tumor/normal brain concentration ratios for cRGD:MID:AC compared with BPA, together with an improvement in rat survival over time. Particularly, new boron compounds combined with cRGD are under development for other tumors like hepatocarcinoma [111]. 

### 3.3. Bimodal Drugs

Bimodal boron drugs, i.e., antitumoral + boron carrier molecules, are under study. For example, Couto et al. [31] studied the effect of sunitinib-decorated carborane hybrids in glioma cells *in vitro*. Icosahedral boron clusters are of interest, as they provide chemical and thermal stability, hydrophilicity/lipophilicity characteristics, and a globular architecture of convenient molecular size to establish interactions. Sunitinib is a second-line chemotherapeutic drug. It is a tyrosine kinase receptor inhibitor, proved useful as an antitumoral and antiangiogenic agent. One of the selected hybrids was found 4 times more cytotoxic than sunitinib and 1.7 times more effective than BNCT mediated by BPA. Then, Alamón et al. [112] proved, in a glioblastoma *in vivo* model, that the therapeutic effect of BNCT mediated by this selected hybrid was significantly higher than that of temozolomide, the most used chemotherapy for glioblastoma, without inducing apparent toxicity effects. 

Temozolomide is a small lipophilic molecule with the ability to penetrate the blood–brain barrier and induce DNA lesions by alkylating the purine bases of DNA. Although it is widely used for treating patients with glioblastoma, approximately half of treated patients exhibit resistance [113]. Xiang et al. [114] proposed a novel strategy to exploit the advantages of TMZ for BNCT: they designed and synthesized a ^10^B-boronated derivative of temozolomide, TMZB. They analyzed boron uptake in three GBM cell lines, U87MG, U251, and HS683GBM, versus BPA. Their results showed that TMZB uptake was cell type-dependent, and among the tested GBM cell lines, U251 cells have the highest uptake when treated with TMZB. However, after BNCT, all cell lines had significantly reduced numbers of surviving clones, and TMZB-based BNCT was significantly more effective than BPA-based BNCT. *In vivo* biodistribution studies showed similar results, as the TMZB-treated group exhibited significantly higher boron concentration versus the BPA-treated group, with a significantly higher tumor/blood and tumor/normal ratio than BPA. BNCT *in vivo* showed higher tumor regression with TMZB-based BNCT compared to the BPA-BNCT group, without significant toxicity. 

A strategy that could take advantage of antitumor immune responses was published by Shi et al. [66]. They studied a carborane-based covalent organic framework (B-COF) to develop a boron “capsule” of immune adjuvants for concurrent BNCT and immunotherapy. COFs are biologically stable and their uniform pore size improves the loading efficiency of drugs and facilitates drug release. In this study, this µm-scale B-COF was used as a boron capsule loaded with a Toll-like receptor (TLR) 7 agonist —imiquimod— combining BNCT with immunotherapy. TLR7 is found on membranes of endosomes and its function is the recognition of nucleosides and nucleotides from intracellular pathogens. The activation of TLR with appropriate agonists induces the innate immune system for defense. Imiquimod is one of the most used agonists in clinical practice and has been approved to treat external genital warts and pre-cancerous skin lesions [115]. It has also been reported as a potential inhibitor of tumor growth that synergizes with radiotherapy [116]. Shi et al. [66] evaluated the cytotoxicity of this boron capsule, B-COF, loaded with imiquimod, on murine melanoma and colorectal cancer cells. They showed that cellular uptake was through endocytosis, and uptake was increased in tumor cells rather than immune cells. They also reported an increase in drug release after neutron irradiation, controlling the immune adjuvant release in the tumor. They showed a significant growth inhibition and membrane damage in both cell lines. Membrane damage is a hallmark for pyroptotic cell death, an inflammatory cell death usually caused by microbial infection. Pyroptosis exerts a tumor suppression function and evokes antitumor immune responses [117]. Based on these observations, they investigated the feasibility of using BNCT mediated by this boron capsule to treat melanoma and induce tumor immunity, in an ectopic immunocompetent mice model. They observed that boron capsules were in the cell cytosol and kept their original morphology, showing an excellent *in vivo* stability. BNCT showed a significant increase in the level of total tumor-infiltrating immune cells and cytokines, turning immunosuppressed tumors into immunogenic tumors. They showed a significant growth inhibition of the primary tumor and distant tumors, i.e., abscopal effect, in melanoma and colorectal xenograft models in mice.

### 3.4. Liposomes and Nanoparticles

Lee et al. [32] showed the potentiality of PEGylated liposomes carrying nido-carboranes to maximize delivery to tumors and minimize uptake in the reticuloendothelial system (RES). Nido-carborane was chosen due to its high boron content per molecule. In an ectopic colon cancer *in vivo* model, they showed an even distribution in tumor tissues and cytoplasm localization in tumor cells. The authors demonstrated significant tumor growth suppression, further enhanced when they performed two BNCT cycles spaced 10 days apart. 

Li et al. [118] described a boronsome (carboranyl-phosphatidylcholine-based liposome) for combinational BNCT and chemotherapy. It accumulates in the tumor due to the permeability and retention (EPR) effect, which may be significantly heterogeneous in patients. Therefore, boronsomes labeled with radioactive nuclides would allow the identification of patients, personalizing BNCT. In this study, they radiolabeled lipids on the bilayer to follow them by PET imaging and loaded them with doxorubicin. In a subcutaneous breast tumor-bearing mouse model, they showed high boron uptake and high ratios of tumor/blood and tumor/normal tissue and demonstrated that BNCT mediated by these boronsomes exhibited a powerful antitumor strategy with good biological safety. They also tested boronsomes loaded with doxorubicin and olaparib. Olaparib is the first poly (ADP-ribose) polymerase-1 (PARP1) inhibitor that has been approved by the FDA. PARP1 is a major enzyme in the repair of DNA strand breaks. Previous studies showed that inhibition of PARP1 increases the efficacy of radiotherapy. They showed, in the same animal model, a synergistic effect of BNCT generating substantial DNA damage and PARP1 inhibitors interfering with DNA repair.

Zaboronok et al. [119] concentrated on the development of elemental boron nanoparticles, or eBNPs, produced from ^10^B microparticles in an aqueous solution and stabilized with a cellulose derivative, hydroxyethylcellulose, approved by the FDA and that meets the requirements of the National Formulary (NF), European Pharmacopoeia (Ph. Eur./EP), and Japanese Pharmacopoeia (JPE). These nanoparticles are incorporated by endocytosis. *In vitro* BNCT experiments in human glioma cell lines were performed to compare the effects of eBNPs to those of BPA. eBNPs significantly reduced colony-forming capacity in all cell lines. 

Wang et al. [120] studied the possibility of combining photothermal therapies with BNCT in colon carcinoma cells and in an *in vivo* model. In that sense, they developed a ^10^B-enriched boron carbide (^10^B_4_C) nanoparticle functionalized with polyglycerol (PG), ^10^B_4_C-PG. Boron carbides can include high ^10^B content and they are inorganic nanoparticles with photothermal effects. Pharmacokinetic experiments showed that ^10^B_4_C-PG had low intrinsic toxicity, boron concentration in the tumor ≥ 20 ppm, and ^10^B concentrations in tumor/blood ≥ 3. ^10^B_4_C-PG-BNCT demonstrated a significant therapeutic effect *in vivo*.

Head and neck cancer is one of the main targets of BNCT. Epidermal growth factor receptor (EGFR) overexpression is an important factor in the pathogenesis of this disease. Moreover, the EGFR-targeting monoclonal antibody cetuximab has been approved for the treatment of late-stage patients [121]. Kuthala et al. [122] designed a ^10^B-enriched ^10^BPO_4_ nanoparticle that was surface-modified with an anti-EGFR antibody. They studied this nanoparticle in head and neck cancer cells and in an *in vivo* model. Anti-EGFR-^10^BPO_4_ NPs/BNCT cytotoxic effects were ~2.4-fold higher compared with BPA-F/BNCT. They demonstrated, compared to BPA-F/BNCT, a significant increase in tumor control and median survival times, together with an effective suppression of tumor recurrences.

### 3.5. Targeting Tumor Cell Metabolism

As we mentioned before, boron molecules that could take advantage of the tumor´s Warburg effect have been studied. The Warburg effect, by which anaerobic glycolysis is upregulated in the growing tumor cells and tissues of malignant tumors, relies on glucose, fructose, galactose, and mannose as the main nutritive supplies. Moreover, GLUT1 transporters are overexpressed in glioma cells. In addition, mannose receptors could also be implicated in the uptake of this carbohydrate, which are expressed in many types of cancer cells. In this sense, Tsurubuchi et al. [123] developed a novel boron compound, based on this knowledge: boron-containing α-d-mannopyranoside (MMT1242), with three closo-dodecarborates, each carrying 12 boron atoms. They demonstrated high uptake, broad intracellular distribution, and longer retention of MMT1242 compared to BSH and BPA, in three different cell lines: melanoma, glioma, and colon tumor cells. Adequate tumor-to-normal tissue accumulation ratio and low toxicity were also demonstrated in an *in vivo* ectopic colon cancer model. BNCT mediated by MMT1242 in this *in vivo* model induced a significant tumor-inhibiting effect. They also studied the possible uptake mechanisms and found that MMT1242 uptake may be GLUT1-independent. They suggested that endocytosis by mannose receptors could be implicated in its incorporation in the tumor. 

### 3.6. Exosomes and Biomimetic Vesicles

The design of new boron compounds was based not only on particular mechanisms and molecules overexpressed in cancer cells. Some examples in the literature were found to be related to exosomes and biomimetic vesicles.

Nanovesicle-shaped particles, called exosomes, are secreted by various cells, including cancer cells. They can function as biomarkers in diagnosis and can modulate the immune system and promote apoptosis, cancer development, and progression. Recently, these nanovesicle particles have attracted attention as potential carriers of natural substances and drugs with anticancer properties and have been proposed as potential boron carriers for BNCT [124]. For example, Hirase et al. [125] developed polyhedral borane anion-encapsulated extracellular vesicles or exosomes, with modification of hexadeca oligoarginine on the exosome membrane. This molecule is a cell-penetrating peptide and is capable of inducing an actin-dependent endocytosis pathway, macropinocytosis. They demonstrated, in glioma cells, an efficient cellular uptake and cancer cell-killing BNCT activity mediated by these boron-loaded exosomes.

Feng et al. [126] reported the possibility of formulating biomimetic vesicles for boron vehiculation. Boron nitride nanospheres were camouflaged with red blood cell membranes. As red blood cells have biomechanical flexibility, non-immunogenicity, inherent biosafety, and immunosuppressive ability to evade phagocytosis, these characteristics could enhance boron compound circulation and stability. Feng et al. [126] demonstrated no *in vivo* toxicity when employing this novel compound strategy, encouraging future BNCT *in vitro* and *in vivo* studies mediated by these biomimetic vesicles.

In Table 2, we summarize all the new boron compounds described in this section.

## 4. Theranostic Boron Compounds to Optimize BNCT

Theranostics is a combination of the terms therapeutics and diagnostics and is a developing new approach to cancer treatment. It pairs diagnostic biomarkers with therapeutic agents to identify and destroy the cancer cells. The principle has been applied in the therapy of thyroid tumors for the last 70 years [127]. Recently, the FDA approved the use of the radiolabeled drug [^177^Lu]Lu-PSMA-617 for the treatment of metastatic prostate cancer [128]. PSMA-617 binds to prostate cancer cells and the ^177^Lu radioisotope emits beta particles that destroy the cancer cells. 

When using a radiopharmaceutical theranostic strategy, the patient is first imaged using a targeted PET or single-photon emission computed tomography (SPECT) tracer, which demonstrates the tracer’s precise distribution and accumulation at the target site, such as a specific receptor that is overexpressed by a particular type of tumor. The patient can then be chosen for endoradiotherapy with a related radiotherapeutical tracer that has the same or a similar structure to the diagnostic tracer, if the relevant tumor and any potential metastases demonstrate sufficient uptake of the labeled biomarker. For BNCT, ideally, the same tracer compound should be both diagnostic and therapeutically useful. A compound that could act as a ^10^B-delivering agent both during therapy and for diagnostic imaging of the macroscopic ^10^B distribution would further advance BNCT. 

**Figure 3:**
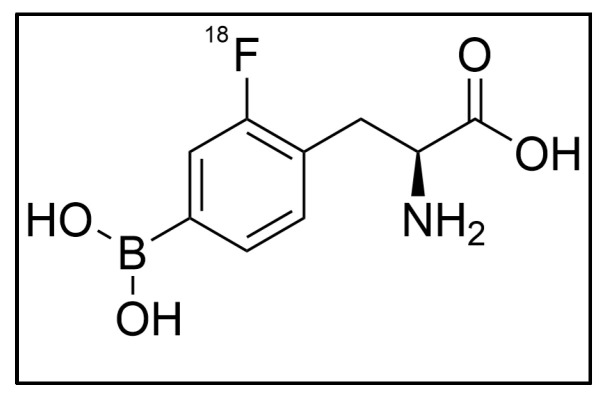
Chemical structure of ^18^F-labeled 2-borono-4-fluoro-L-phenylalanine (^18^F-BPA) marker.

One of the most widely used imaging methods to perform functional diagnosis of malignant tumors is using a PET scan along with a ^18^F-fluorodeoxyglucose (FDG) marker. This method is used to examine the stage, therapeutic effect, and recurrence of malignant tumors by utilizing the property that malignant tumor cells take up 3–8 times more glucose than normal cells [129]. However, this imaging modality also detects inflammatory activity [130], thereby making it difficult to distinguish between malignant tumors from inflammatory lesions. A similar method is using a ^18^F-labeled 2-borono-4-fluoro-L-phenylalanine (^18^F-FBPA) marker (Figure 3). Its uptake reflects amino acid metabolism because ^18^F-FBPA, a phenylalanine boronated molecule, behaves similarly to cells’ intrinsic phenylalanine. Malignant tumor cells have a higher rate of metabolism, and ^18^F-FBPA accumulates specifically in malignant tumors [131,132], whereas physiological accumulation of ^18^F-FBPA is minimal in normal organs, with the exception of the urinary system [133,134]. 

For the assessment of tumor uptake of boron (^10^B) during BNCT for refractory and recurring head and neck cancer and brain tumors, ^18^F-FBPA-PET has been used in clinical studies [135,136]. A recent study performed by Isohashi et al. [137] investigated the uptake of boron inside both malignant and benign lesions of 82 patients with various types of cancer. The results showed the maximum standardized uptake value for malignant tumors to be significantly higher than that of benign lesions. This information is useful when determining whether a patient is suitable for BNCT and accurate determination of the dose delivered to the tumor. However, there are several limitations of ^18^F-FBPA. Firstly, F-BPA PET provides tracer dose pharmacokinetics of F-BPA, while in BNCT therapeutic doses of BPA are administered (approximately 500 mg/kg). Secondly, BPA is administered by slow bolus intravenous injection followed by drip infusion during BNCT irradiation, while F-BPA is administered by a single bolus injection. These variations make it unclear whether F-BPA PET has any predictive value for BPA accumulation in tumor and healthy tissues. Furthermore, ^18^F-FBPA is not yet an approved imaging modality, therefore not all patients that undergo BNCT can receive it. Hopefully, in the near future, this imaging method will be approved and all patients that undergo BNCT can receive it.

Boron uptake and microdistribution in the tumor vary among patients. Historically, tumor boron concentration in clinical trials has been estimated using empirical data models. In this sense, imaging methods to monitor, in real time, boron uptake and distribution for each patient and each tumor would help to personalize BNCT and improve therapeutic outcomes, with less associated toxicity [138,139,140].

Several of the abovementioned discussed developments on new boron compounds have been modified to be useful as theranostic compounds, e.g., [102,118]. Apart from these, more examples can be found in the literature although they have not been tested in BNCT radiobiological studies yet (e.g., [141,142,143,144]). 

For example, Freiner et al. [145,146] took an approach where a tumor-targeting trans-cycloocetene (TCO)-functionalized monoclonal antibody (mAb) was initially administered followed by an injection of tetrazine (Tz)-functionalized boron-rich carbon-based nanoparticles. The reaction between the TCO-mAb and the Tz-functionalized nanoparticles resulted in an enhanced tumor retention when compared to the nanoparticles alone. Among various candidates, boron-rich gold nanoparticles, due to the chemically inert and non-toxic properties, were selected and evaluated as a pre-targeting strategy. *In vivo* studies in tumor-bearing animals confirmed the accumulation of the nanoparticles in the tumor with significant uptake. A pre-targeting strategy enhanced the accumulation of boron-rich nanoparticles and showed potential application in BNCT. 

In the case of Pulagam et al. [147], they developed gold nanorods (AuNRs) stabilized with polyethylene glycol and functionalized with the water-soluble complex cobalt bis(dicarbollide) –COSAN- ([3,3′-Co(1,2-C_2_B_9_H_11_)^2^]^−^). Radiolabeling with the positron emitter copper-64 (^64^Cu) enabled *in vivo* tracking using positron emission tomography imaging. ^64^Cu-labeled multifunctionalized AuNRs proved to be radiochemically stable and capable of being accumulated in the tumor after intravenous administration in a mouse xenograft model of gastrointestinal cancer. 

Torresan et al. [140] investigated the use of iron–boron nanoparticles to assist neutron capture therapy with the use of magnetic resonance imaging (MRI). The magnetic properties of the nanoparticles are also of interest for magnetophoretic accumulation in tissues and magnetic hyperthermia to assist drug permeation in tissues. This represents a promising tool for future MRI-guided BNCT. 

Kanygin et al. [148] used liposomes with a fluorescent label (Nile Red) in both *in vitro* and *in vivo* experiments in a bid to develop a low-cost and rapid verification of liposomes accumulating in the target cells. The results of this study showed fluorescence microscopy can be used as an effective, rapid method to determine the intracellular localization of liposomes with a fluorescent label. 

Deng and Yu [149] put together a summary of the recent development of radiofluorination of boron agents for BNCT. A trifluoroborate-containing amino acid such as fluoroborontyrosine (FBY) could be a potential candidate for BNCT that has both functionalities of imaging and therapeutics. Similarly to BPA, FBY showed low toxicity with high tumor/normal ratio from the viewpoint of diagnosing gliomas. However, the concentration of boron inside the glioma has not yet been provided. Despite the several advantages of FBY, more evidence and animal studies are required before it can be used in clinical trials of BNCT. 

Kondo et al. [150] developed a novel fluorescence boron sensor (BS-631) which emits fluorescence with a maximum wavelength of 631 nm after the boron neutron capture reaction. By detecting this fluorescence, the localization of the BPA within the cells can be determined. Several other authors have investigated the labeling of boron compounds with a fluorescence marker. Preliminary results indicate a positive step forward toward optimizing BNCT, however, when preparing on a larger scale (i.e., human beings) the compound appears to be quite toxic for clinical applications. The discovery of potential theranostic candidates for BNCT still poses a challenge for researchers [142]. 

## 5. Conclusions and Future Directions

It is well known that if an “ideal” boron carrier is identified as promising, many years of studies and very high costs will be necessary for it to be approved for treatment [151]. However, if it is not now…when? Huge efforts have been made in the development of accelerators for BNCT and clinical trials using these devices for treating patients [152,153]. Perhaps it is coming to the time to dive into this huge amount of work devoted to the development of new boron compounds and take the next step: introduce them in the clinical field. Meanwhile, those strategies that are approved for humans, which were demonstrated as useful to increase the therapeutic outcome of BNCT mediated by the well-known boron compounds BPA, BSH, GB-10, and BA, will increase patient survival and quality of life, in a time- and cost-effective way [47,151,154].

Recent advances in omics technologies could help on the design of personalized boron compounds [155]. Omics technologies and computational tools will be merged in a future strategy to enable the development of so-called systems medicine. Genomics, transcriptomics, epigenomics, proteomics, metabolomics, and other omics areas have contributed to discovering and describing key mechanisms in cancer development, treatment resistance, and recurrence risk [156]. Each tumor has its own characteristics, with particular gene mutations and with a tumor microenvironment that could vary in terms of oxygen availability, vascularity, etc. Omics technologies can molecularly characterize tumors and physiopathological conditions and would contribute on the design of new boron compounds and strategies aimed at particular characteristics of the tumor [155]. The design of new boron compounds targeting these newly described molecules and mechanisms will significantly improve BNCT for the well-studied pathologies but also extend BNCT to unexplored tumors. Omics technologies could help to guide BNCT decisions in terms of which boron compound has to be used and personalizing dose planning [155]. 

Radiobiological studies are important to decide if new boron compounds, derivatives, and strategies are therapeutically useful and could be translated to the clinical field. We explored those publications, between 2020 and 2023, in which BNCT *in vitro* and *in vivo* radiobiological studies of new derivatives of BPA, BSH and BA, strategies combined with BNCT and new boron compounds solved the limitations associated to the uptake, microdistribution and retention of boron compounds in the tumor, or act as anticancer strategies enhancing even more BNCT therapeutic effect. However, there are also very important publications, between 2020 and 2023, in which the authors demonstrated how tumor characteristics would negatively condition BNCT’s therapeutic effect combined with well-known and approved therapies. Sanada et al. [64] reported that hypoxia decreases the effect of BPA/BNCT but not BSH/BNCT, as LAT1 is downregulated in this condition. For example, Kinashi et al. [157] reported that DNA repair enzyme O6-methylguanine DNA methyltransferase (MGMT) and tumor suppressor p53 mutations in glioblastoma cells could affect the cell-killing effect of temozolomide combined with BNCT. Another example was published by Tatebe et al. [158] studying BNCT combined with rapamycin. The mammalian target of rapamycin (mTOR) signaling pathway is implicated in resistance to therapy and poor treatment outcomes. Rapamycin inhibits this pathway and thus tumor growth. However, rapamycin can also inhibit BPA uptake, potentially affecting BPA/BNCT’s therapeutic effect on tumors. 

In this review, we analyzed new boron compound radiobiological studies (*in vitro* and *in vivo*) that were published between 2020 and 2023. In some publications, authors showed that the therapeutic outcome of BNCT compared to BNCT mediated by BPA or BSH was improved. It has been reported that boron compounds for use in clinical BNCT must exhibit the following principal properties [9,25]: the distribution ratio for tumor to healthy cells must be higher than 3:1; the tumor uptake should be approximately 20–50 μg of ^10^B per g; there should be no pharmaceutical effects and it must function only as a boron delivery molecule; it must remain in the tumor cell for a few hours; rapid clearance of boron compound from healthy cells; low toxicity since it is administered directly into the blood; appropriate water solubility. These characteristics were studied in the reported publications and were considered in the design of new boron compounds. With recent advancement in accelerator technology, a higher neutron flux than the reactor-based neutron sources can be achieved, and the treatment time will be shorter. This kind of information may help researchers that are developing boron compounds, as it will loosen some of the criteria mentioned above.

Clinical trials, which provide scientific evidence of the safety and effectiveness of novel pharmaceutical compounds and advise clinical care, are a crucial component of drug development. However, apart from the high economic costs, one of the major challenges when performing a clinical trial is patient recruitment. Given the strict criteria, and also the fact that BNCT is still not widely recognized by many clinical institutes, the recruitment process takes a very long time. There is also a physical challenge, where patients cannot travel to the clinical trial site for treatment/follow up on a regular basis, may which degrade the clinical trial results. Fogel et al. [159] stated that traditional clinical trials are slow, expensive, and inefficient, which is exactly what was noticed when performing the phase II clinical trial using an accelerator-based BNCT system for refractory recurrent high-grade meningioma [160]. Stakeholders of clinical trials are increasingly realizing that in order to overcome these obstacles and meet patients’ requirements, a radical overhaul of the way conventional clinical trials are carried out is required. One potential method to overcome the above challenges is utilizing a virtual clinical trial [161]. These virtual clinical trials can take advantage of digital health technologies to gather data at each stage of the clinical trial, increase patient recruitment and retention, enable online informed consent, measure real-time clinical endpoints, and continuously monitor adverse events. This new model will allow patients to participate from their homes rather than traveling to the clinical trial site, which may potentially increase the number of participants.

Other possible limitations are the uncertainties related to BNCT dosimetry and treatment planning [26]. For example, BPA is assumed to be uniformly taken up inside the cell. If a new compound can be selectively taken up inside the cell (non-uniform distribution), this information also needs to be fed into the treatment planning system for physicians to understand the treatment outcome. The development of new theranostic compounds is an active area that will help in this field, as it enables the online study of boron uptake and distribution in the tumor and organs of interest [162]. Moreover, the design of new treatment planning systems (e.g., [163]) that could consider the features of each boron compound (known by biodistribution, microdistribution, and theranostic studies) would give a big jump in this field, not only for clinical studies but also to increase the efficiency in radiobiological studies. 

There is a lot to explore. New advances in cancer mechanisms and discoveries related to the different hallmarks of cancer [164] should be considered in the design of new strategies and boron compounds. One example is targeting tumor cell metabolism, considered a hallmark of cancer that was found to be related to tumor immunity and can be exploited in cancer treatment [164,165,166]. Regulated cell death plays a crucial role in cancer metabolic therapy. A recent study has identified a new type of metabolic-related regulated cell death known as disulfidptosis. Pre-clinical findings suggest that metabolic therapy using glucose transporter (GLUT) inhibitors can trigger disulfidptosis and inhibit cancer growth [166]. Boron compounds that could target GLUT1 transporters would increase BNCT’s therapeutic effect. Moreover, studies have shown that glucose metabolism inhibition promotes compensatory LAT1 upregulation, possibly enhancing BPA uptake [167]. In that sense, the combination of BPA with these boron compounds would be an interesting approach to enhance boron uptake and BNCT’s therapeutic effect. 

Evading immune system destruction is another hallmark of cancer [164]. The recent discovery of cancer cell-intrinsic surface-expressed programmed death ligand 1 (PDL1) signals to immune cell programmed death 1 (PD1) to inhibit antitumor immunity contributed to developing revolutionary immunotherapies. PDL1 is an immune checkpoint molecule that regulates T cell proliferation and interleukin secretion. It is expressed in normal and cancer cells. In tumors, PDL1 interacts with PD1 on T cells, leading to the inhibition of T cell activation (including T cell apoptosis), consequently inhibiting antitumor immunity. It was demonstrated that antibodies blocking PDL1 or PD1 improve prolonged survival and antitumor immunity in animal cancer models and humans. Another important immune checkpoint is cytotoxic T lymphocyte-associated antigen-4 (CTLA4), also upregulated in activated T cells. Several immune checkpoint blockade agents, blocking PDL1 and CTLA4 immune checkpoints, are approved by the FDA as cancer immunotherapies [168,169]. These approved agents could be designed to carry boron atoms, to combine this strategy with BNCT, increasing therapeutic effects and overcoming limitations of both therapies. In relation to this topic, a recent study by Mohammed et al. [170] with boron derivatives (sodium pentaborate pentahydrate (SPP) and sodium perborate tetrahydrate (SPT)) demonstrated that these compounds induced proliferation suppression and apoptosis in two human breast cancer cell lines. However, these boron compounds induced stimulatory effects on the PD1/PDL1 signaling pathway. 

The tumor microbiota has been included as one of the hallmarks of cancer in the recently published review of Hanahan [164]. Microorganisms, like fungi and different species of bacteria (commensal and opportunistic) and viruses, within the gut, oral cavity, skin, and other organs may contribute to carcinogenesis, as well as shaping cancer immunosurveillance and response to antitumor strategies. Different factors can alter the proportion and composition of this microbiota and, if this alteration increases the abundance of pathogen species or reduces the beneficial ones, this could initiate disease. One of these factors could be cancer therapy. For example, it has been stated that there is a bidirectional interaction between microbiota and ionizing radiation in head and neck cancer. Head and neck cancer patients show radiation-induced toxicities, like xerostomia and mucositis, that could lead to disruptions in the microbial colonies that in turn enhance further adverse effects [171,172]. Moreover, Hu et al. [173] reported that oral microbiota richness negatively correlates with radiation doses in head and neck cancer patients treated with radiotherapy. Strategies that could modulate oral microbiota could enhance cancer therapy’s effect on tumors and help to reduce toxicity [171]. Probiotics (live microorganisms to treat specific diseases by improving microbial flora), prebiotics (nutrients that are degraded by microbiota), or antibiotics showed that it is possible to select those strains that could protect the patient against cancer development and inflammation after treatment [174,175,176]. Moreover, for example, some prebiotics have been reported as an adjunct to cancer therapies [177,178,179]. Mucositis and xerostomia are induced by BNCT and limit the dose to the tumor, apart from affecting the quality of life in head and neck cancer patients (e.g., [180]). In this sense, the impact of BNCT on tumors and normal tissue/organ microbiota should be explored. New strategies combining BNCT with prebiotics, probiotics, and antibiotics approved for use in humans could be interesting strategies to increase tumor control and reduce radiotoxicity in dose-limiting tissues. 

Chimeric antigen receptor T cell (CAR-T) therapy is a revolutionary cellular therapy, with effective and durable clinical responses. CAR-engineered synthetic receptors redirect lymphocytes (mostly T cells) to recognize and eliminate cells expressing a specific target antigen, for example, specific tumor antigens. CAR-T therapy is approved for the treatment of hematological malignancies, however, it exhibits several challenges, e.g., limited effect on solid tumors [181]. Seneviratne et al. [26] explained that radiotherapy can mitigate those challenges and, particularly, high-LET particles would increase this effect. They explained that the increase in cytotoxicity also increases the elaboration of antigens and some radiation-induced neoantigens in irradiated cancer cells could be targets for CAR-T cells. As high-LET particles are also able to destroy radioresistant hypoxic cancer cells, CAR-T cells would also be useful to reach resistant cells in deep recesses of the tumor with a hostile microenvironment. Moreover, a proinflammatory immune microenvironment induces cytokine- and chemokine-mediated chemotaxis on CAR-T cells. Due to all these phenomena, they suggest that CAR-T cells may be potentially useful vehicles to selectively deliver boron to tumor cells, enhancing BNCT’s selectivity and therapeutic effect.

Finally, in the field of multimodal radiotherapies to improve antitumoral outcomes, a recent publication by Nunez Martinez et al. [182] reported significant cell damage using ferrabis(dicarbollides) to perform proton boron fusion radiation therapy (PBFRT). PBFRT is based on the interaction between a proton and ^11^B that generates three high-LET alpha particles, increasing significantly the dose delivered to the tumor [183].

This article was written by a BNCT radiobiologist and a BNCT medical physicist. This article shows how questions that arise from the clinical field can be answered by radiobiological experiments, and how new discoveries in the radiobiological field would improve significantly BNCT’s therapeutic effect in humans. We also discussed the limitations that have to be overcome to introduce these recent advances that would increase significantly BNCT’s therapeutic effect and patient survival and quality of life. In line with Bortolussi et al. [184], we consider that active communication between radiobiology, clinics, and all disciplines is fundamental to push BNCT forward. 

## Figures and Tables

**Table 1:** BPA-, BSH-, GB-10-, boric acid-mediated BNCT, derivatives, and strategies: BNCT *in vivo* and *in vitro* radiobiological studies.

Boron Compound/Strategy	Pathology	References
BPA/BSH/GB-10/Boric Acid and Derivatives		
BPA	Cervical cancer cells Pelvic CRC (*in vivo*) PCNSL (*in vitro* and *in vivo*) Bone metastasis (breast cancer) (*in vivo*)	[48] [50] [51] [56]
BPA + low-dose of γ-radiation (0.1 Gy)	Squamous cell carcinoma orthotopic model	[61]
BPA + radiosensitizer (NaB)	Ectopic thyroid cancer model	[62]
BPA + YC-1 (HIF-1α-targeting inhibitor)	Head and neck cancer and glioblastoma cells	[63]
BPA + BCG (bacillus Calmette–Guérin—immunotherapy agent)	Ectopic colon cancer model	[69]
BPA–amide alkyl dodecaborate (convention enhanced delivery)	Glioma, melanoma, and squamous carcinoma (*in vitro* and *in vivo*)	[33]
Poly(vinyl alcohol)-BPA	Ectopic pancreatic adenocarcinoma and colon cancer	[38]
Mesoporous organosilica nanoparticles loaded with BPA and BSH	Ovarian cancer cells and spheroids Chicken egg human ovarian tumor model	[39] [72]
BSH-Z33 peptide–cetuximab (EGFR targeting)	*In vitro* human epidermoid carcinoma	[76]
^10^BSH-entrapped transferrin-conjugated polyethylene glycol liposome constructed with distearoyl-boron lipids (BSH-TF-PEG-DSBL)	*In vivo* hepatic tumor model	[78]
Poly-arginine peptide conjugated with BSH (CD44 cell surface and translation-related protein targeting)	Glioma, breast and pancreatic cancer cells	[79]
A6K/BSH (peptide nanotubes—siRNA delivery tool)	Glioma cells	[80]
GB-10 + electroporation	Oral cancer *in vivo* model	[83]
Boric acid	Osteosarcoma *in vivo* model Hepatocellular carcinoma cells	[84] [86]
IF7C(^10^BPA)RR or IF7K(^10^BSH)RR (annexin A1 targeting)	Bladder cancer	[55]
Boric acid-containing chitosan/alginate/polyvinyl alcohol nanoparticles (BA-capNPs)	Oral squamous cell carcinoma cells	[87]
Boric acid–styrene–maleic acid copolymer (SMA) conjugated with glucosamine (SG) complex	Oral cancer cells Squamous cell carcinoma *in vivo* model	[88]

**Table 2:** New boron compounds proved therapeutically useful for BNCT in *in vitro* and *in vivo* models.

Boron Compound/Strategy	Pathology	References
**Targeting structures and relevant molecules**		
DOXCB@lipo-pDNA-iRGD multifunctional nanoliposome: boron neutron capture immuno-chemotherapy (DNA targeting via doxorubicin)	Glioma *in vivo* model	[6]
DPA-BSTPG: dodecaborated compound targeting TSPO mitochondria protein +/− BPA (mitochondria targeting via TSPO proteins)	Glioblastoma *in vitro* and *in vivo*	[102]
Carboranylmethylbenzo[b]acridone (CMBA) (cytoskeleton and membrane targeting)	Glioblastoma cells	[103]
**Nanobiocarrier: albumin**		
Maleimide-functionalized closo-dodecaborate conjugated to albumin (MID:albumin) cRGD:MID:albumin (cRGD: target tumor cells and tumor neovascularization)	Orthotopic glioblastoma model Chemically induced oral cancer model Subcutaneous glioblastoma and ectopic colon cancer models Orthotopic glioblastoma model	[41] [107] [108] [110]
**Bimodal drugs**		
Sunitinib-decorated carborane hybrid	Glioblastoma *in vitro* and *in vivo*	[31] [112]
^10^B-boronated derivative of temozolomide	Glioblastoma *in vitro* and *in vivo*	[114]
Boron capsule: carborane-based covalent organic framework (B-COF) loaded with imiquimod Imiquimod: Toll-like receptor (TLR) 7 agonist with antitumoral and immunmodulatory activities—BNCT + immunotherapy	Melanoma and colorectal cancer cells Ectopic melanoma model	[66]
**Liposomes and nanoparticles**		
PEGylated liposomes carrying nido-carboranes	Ectopic colon cancer model	[32]
Boronsome (carboranyl-phosphatidylcholine-based liposome) loaded with doxorubicin and olaparib—BNCT + chemotherapy	Subcutaneous breast tumor model	[118]
eBNPs: elemental boron nanoparticles	Glioma cells	[119]
Boron carbides—^10^B_4_C-PG: ^10^B-enriched boron carbide (^10^B_4_C) nanoparticle functionalized with polyglycerol (PG) Photothermal therapy + BNCT	Colon carcinoma model (*in vitro* and *in vivo*)	[120]
^10^B-enriched ^10^BPO_4_ nanoparticle surface-modified with an anti-EGFR antibody	Head and neck cancer (*in vitro* and *in vivo*)	[122]
**Targeting tumor cell metabolism**		
MMT1242: boron-containing α-d-mannopyranoside (endocytosis by mannose receptors)	Melanoma, glioma, and colon tumor cells Ectopic colon cancer model	[123]
**Exosomes and biomimetic vesicles**		
Polyhedral borane anion-encapsulated exosomes, with modification of hexadeca oligoarginine on the exosome membrane (macropinocytosis) Boron nitride nanospheres covered with red blood cell membranes (biomimetic vesicles). No BNCT *in vitro*/*in vivo* studies.	Glioma cells Human embryonic kidney and human cervical carcinoma cell lines (HeLa cells). *In vivo* toxicity study in mice (no tumor). No BNCT studies	[125] [126]
